# Randomized Controlled Trials in ICU in the Four Highest-Impact General Medicine Journals

**DOI:** 10.1097/CCM.0000000000005937

**Published:** 2023-05-18

**Authors:** Jasper M. Kampman, Nicolaas H. Sperna Weiland, Jeroen Hermanides, Markus W. Hollmann, Sjoerd Repping, Janneke Horn

**Affiliations:** 1 Department of Anesthesiology, Amsterdam UMC, Amsterdam, The Netherlands.; 2 Department of Intensive Care Medicine, Amsterdam UMC, Amsterdam, The Netherlands.; 3 Department of Health Evaluation and Appropriate Use, Amsterdam UMC, Amsterdam, The Netherlands.; 4 National Healthcare Institute, Diemen, The Netherlands.

**Keywords:** bias, clinical trials, critical care, Fragility Index, funding, intensive care, sample size

## Abstract

**OBJECTIVE:**

To study ICU trials published in the four highest-impact general medicine journals by comparing them with concurrently published non-ICU trials in the same journals.

**DATA SOURCES:**

PubMed was searched for randomized controlled trials (RCTs) published between January 2014 and October 2021 in the *New England Journal of Medicine*, *The Lancet*, the *Journal of the American Medical Association*, and the *British Medical Journal.*

**STUDY SELECTION:**

Original RCT publications investigating any type of intervention in any patient population.

**DATA EXTRACTION:**

ICU RCTs were defined as RCTs exclusively including patients admitted to the ICU. Year and journal of publication, sample size, study design, funding source, study outcome, type of intervention, Fragility Index (FI), and Fragility Quotient were collected.

**DATA SYNTHESIS:**

A total of 2,770 publications were screened. Of 2,431 original RCTs, 132 (5.4%) were ICU RCTs, gradually rising from 4% in 2014 to 7.5% in 2021. ICU RCTs and non-ICU RCTs included a comparable number of patients (634 vs 584, *p* = 0.528). Notable differences for ICU RCTs were the low occurrence of commercial funding (5% vs 36%, *p* < 0.001), the low number of RCTs that reached statistical significance (29% vs 65%, *p* < 0.001), and the low FI when they did reach significance (3 vs 12, *p* = 0.008).

**CONCLUSIONS:**

In the last 8 years, RCTs in ICU medicine made up a meaningful, and growing, portion of RCTs published in high-impact general medicine journals. In comparison with concurrently published RCTs in non-ICU disciplines, statistical significance was rare and often hinged on the outcome events of just a few patients. Increased attention should be paid to realistic expectations of treatment effects when designing ICU RCTs to detect differences in treatment effects that are reliable and clinically relevant.

**KEY POINTS**

**Question**: How do ICU randomized controlled trials (RCTs) published in the *New England Journal of Medicine*, *The Lancet*, the *Journal of the American Medical Association*, and the *British Medical Journal* compare with concurrently published non-ICU RCTs?

**Findings**: Of 2,431 original RCTs, 132 (5.4%) were ICU RCTs. Notable findings included that ICU RCTs were as large as non-ICU RCTs, that commercial funding was rare for ICU RCTs, and that most ICU RCTs did not report statistically significant findings. When significance was reached, the results were generally fragile and hinged on just a few outcome events.

**Meaning**: RCTs in ICU medicine made up a growing portion of RCTs published in high-impact general medicine journals. Compared with RCTs in other disciplines, increased attention should be paid to realistic expectations of treatment effects to detect reliable and clinically relevant differences.

Randomized controlled trials (RCTs) are the gold standard in evidence-based medicine. The validity and usefulness of RCTs are determined by a multitude of factors that are often debated (1). Common problems are a limited sample size, an unsuitable choice of outcome measure, or fragility of the results (2, 3). In ICU medicine most landmark trials are not published in ICU specialty journals, but published in high-impact general medicine journals (4). The aim of this meta-research study was to describe the main characteristics of contemporary ICU RCTs in these top journals, and compare them with concurrently published non-ICU RCTs.

## METHODS

The protocol of the data collection for the current study was registered with the Open Science Framework (DOI: 10.17605/OSF.IO/PEKSX). The Preferred Reporting Items for Systematic Reviews and Meta-Analyses guidelines were used for the design and reporting (5).

We searched PubMed for RCTs published between January 1, 2014, and October 1, 2021, in the top four medical journals based on impact factor (**Appendix 1**, http://links.lww.com/CCM/H350), which were the *New England Journal of Medicine*, *The Lancet*, the *Journal of the American Medical Association*, and the *British Medical Journal*. These high-impact journals were chosen because of their wide scope, broad audience, and influential position in guiding clinical practice. All original RCTs investigating any type of intervention in any patient population were included. Follow-up trials and interim analyses were excluded to prevent a trial from appearing twice in our database (**Appendix 2**, http://links.lww.com/CCM/H350). Trials were defined as ICU RCTs if they exclusively included patients admitted to the ICU. Collected characteristics were year and journal of publication, sample size, study design, funding source, study outcome, type of intervention, the Fragility Index (FI), and Fragility Quotient (FQ). The FI is a metric that calculates the number of outcome events on which statistical significance is dependent. It is calculated by converting one patient in the group with the smallest number of events from a nonevent outcome to an event outcome and recalculating a two-sided Fisher exact test. This is repeated until the *p* value meets or exceeds 0.05. It can only be calculated for statistically significant, superior RCTs that use a binary primary outcome. We used a free and reproducible online FI calculator (6). When an RCT compared more than two groups, we calculated the various intergroup FIs and selected the highest to avoid overestimating statistical fragility. We calculated the FQ by dividing the FI by the sample size. The FQ reports the fraction of the sample size on which statistical significance is dependent. Data were extracted independently by two reviewers (J.M.K., J.H.). Complete eligibility criteria and characteristics are listed in Appendices 2 and **3** (http://links.lww.com/CCM/H350).

## RESULTS

**Figure 1.**
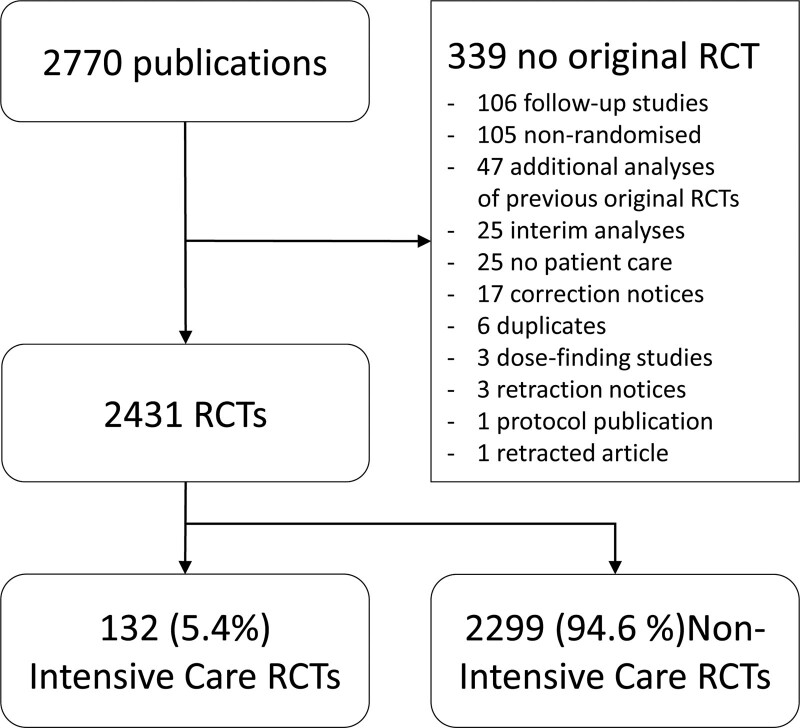
Preferred Reporting Items for Systematic Reviews and Meta-Analyses flow diagram of study inclusion. RCT = randomized controlled trials.

A total of 2,431 RCTs were included, of which 132 (5.4%) were ICU RCTs (**Fig. 1**). This percentage gradually rose from 4% in 2014 to 7.5% in 2021 (**Table 1**). The median sample size of ICU RCTs was 634 (IQR: 311–1,664) compared with non-ICU RCTs (584, IQR: 256–1,645, *p* = 0.528). The number of positive RCTs (i.e., reached statistical significance for the primary outcome) was lower for ICU RCTs than for non-ICU RCTs (29% vs 65%, *p* < 0.001). Industry funding was rare among ICU RCTs, with just 5% of trials receiving exclusively commercial funding, compared with 36% for non-ICU trials (*p* < 0.001). A noninferiority design was uncommon in ICU RCTs (4% vs 13%, *p* < 0.001).

**TABLE 1.** Characteristics of Contemporary ICU Randomized Controlled Trials and Non-ICU Randomized Controlled Trials in High-Impact Journals

Characteristic	ICU RCTs (*n* = 132)	Non-ICU RCTs (*n* = 2,299)	*p*
Journal			
*New England Journal of Medicine*	48 (36%)	957 (42%)	0.239
*The Lancet*	8 (6%)	706 (31%)	< 0.001
*Journal of the American Medical Association*	71 (54%)	488 (21%)	< 0.001
*British Medical Journal*	5 (4%)	148 (6%)	0.271
Sample size			
Median (IQR)	634 (311–1,664)	584 (256–1,645)	0.528
Year of publication			
2014	12 (4%)	288	
2015	15 (4.5%)	322	
2016	18 (6%)	280	
2017	11 (3.6%)	296	
2018	21 (6.5%)	301	
2019	19 (5.5%)	329	
2020	19 (6.5%)	272	
2021 till October 1	17 (7.5%)	211	
Type of funding			
Commercial	6 (5%)	825 (36%)	< 0.001
Institutional	106 (80%)	1,144 (50%)	< 0.001
Both	17 (13%)	313 (14%)	0.896
Neither	3 (2%)	17 (1%)	0.091
Study design			
Superiority	127 (96%)	2,007 (87%)	< 0.001
Noninferiority	5 (4%)	292 (13%)	< 0.001
Type of intervention			
Medicine/fluids/food	77 (58%)	1,549 (67%)	0.036
Device/material	27 (20%)	144 (6%)	< 0.001
Surgery/procedure	7 (5%)	269 (12%)	0.023
Behavioral/training	7 (5%)	198 (9%)	0.257
Organizational	12 (9%)	46 (2%)	< 0.001
Diagnostic	2 (2%)	92 (4%)	0.239
Study outcome			
Statistically significant (“positive”)	38 (29%)	1,500 (65%)	< 0.001
Statistical fragility			
RCTs eligible	19	604	
Fragility Index	3 (IQR: 1–6)	12 (IQR: 4–29)	0.008
Fragility Quotient	0.006 (0.002–0.018)	0.015 (0.004–0.045)	0.063

IQR = interquartile range; RCT = randomized controlled trials.

Fisher exact test was used for ordinal data and an independent-samples median test was used for continuous data.

The median FI of ICU RCTs was much lower than for non-ICU RCTs (3 events [interquartile range, IQR: 1–6] vs 12 events [IQR: 4–29], *p* = 0.008). The FQ was also lower for ICU RCTs but this difference did not reach statistical significance (0.006 [IQR: 0.002–0.018], vs 0.015 [IQR: 0.004–0.045], *p* = 0.063). **Appendix 4** (http://links.lww.com/CCM/H350) lists all ICU RCTs eligible for the FI analysis, including the trial’s primary outcome measures, which were mortality-based in 26% of RCTs. Other primary outcomes included rates of infection, (re)intubation, or composite outcomes.

## DISCUSSION

In the last 8 years, RCTs in ICU medicine made up a meaningful portion of RCTs published in high-impact journals, doubling from 4% in 2014 to 7.5% in 2021. A notable similarity between ICU and non-ICU RCTs was the comparable number of patients included. Clear differences included the low occurrence of commercial funding for ICU RCTs, the low number of RCTs that reached statistical significance, and the low FI when they did.

We believe the publication of negative results should be strongly advocated. Therefore, our finding that only 29% of ICU RCTs report statistical significance is in itself not worrisome. At the same time, when RCTs did reach statistical significance, the results were much more fragile than concurrent non-ICU RCTs according to the FI. These findings match a recent umbrella review that showed a median FI of 2.5 (IQR: 1–5.5) for RCTs published in ICU specialty journals (7). The FI is easy to understand metric that can add extra insight to the *p* value, instead of just reporting whether the arbitrary cutoff of 0.05 was reached. However, it has also received criticism. A low FI could penalize small trials with impressive results, whereas a high FI could falsely suggest that this is the same as clinical relevance (8). The FI tries to summarize in a single number the complex relationship among sample size, effect size, and *p* value. Therefore, the FI should not be used as a standalone metric to compare trials (9). In the context of the RCT from which it was derived, we believe the FI adds valuable information to reported results and *p* values.

In the current study, the FI of RCTs in ICU medicine was much lower while the sample size was comparable, suggesting either low effect sizes or low outcome incidences. This would fit a recent publication showing that ICU RCTs were often powered for overly optimistic effects and routinely overestimated mortality in the control group (10). Half the included trials were powered for a reduction of 10% in absolute mortality, which was only reached in a few single small trials. Another publication described similar results, showing that an average mortality reduction of 10% was used in sample size calculations, while the eventually demonstrated mortality reduction was 10-fold lower, around 1% (11). These overestimations thwart the sample size calculation, and could explain our findings of trials that do not, or just barely, reach statistical significance.

Simultaneously, the advice to increase the sample size of RCTs comes with its own difficulties. It will often require adding centers, increasing costs, and the chance of practice heterogeneity. For ICU interventions, adoptions are often based on just one or two trials, so their reliability is crucial. Ultimately, trials should be powered for the smallest effect size that is clinically important and should not be missed.

Caring for the critically ill is complex, and improving ICU outcomes is difficult. This stresses the importance of adequately powered trials with realistic expectations regarding the effect of the studied intervention. Our results suggest that this can be improved in future ICU RCTs.

A possible limitation of our study is that the inclusion period contains part of the COVID-19 pandemic in 2020 and 2021. RCTs in these years might have been influenced by the aim of finding effective therapies and vaccines. Of the 2,431 included RCTs, we found that 60 (2%) covered COVID-19. For the ICU RCTs, 8 of 132 trials covered COVID-19. We believe these small numbers had no relevant impact on our conclusions.

## CONCLUSIONS

In the last 8 years, RCTs in ICU medicine made up a meaningful, and growing, portion of RCTs published in high-impact general medicine journals. In comparison with concurrently published RCTs in non-ICU disciplines, statistical significance was rare and often hinged on the outcome events of just a few patients. Increased attention should be paid to realistic expectations of treatment effects when designing ICU RCTs.

## Supplementary Material



## References

[1] Macleod MR Michie S Roberts I: Biomedical research: Increasing value, reducing waste. Lancet. 2014; 383:101–104 24411643 10.1016/S0140-6736(13)62329-6

[2] Gaudino M Hameed I Rahouma M: Characteristics of contemporary randomized clinical trials and their association with the trial funding source in invasive cardiovascular interventions. JAMA Intern Med. 2020; 180:993–1001 32478821 10.1001/jamainternmed.2020.1670 PMC7265124

[3] Demarquette A Perrault T Alapetite T: Spin and fragility in randomised controlled trials in the anaesthesia literature: A systematic review. Br J Anaesth. 2023; 130:528–535 36759291 10.1016/j.bja.2023.01.001

[4] Pensier J De Jong A Chanques G: A multivariate model for successful publication of intensive care medicine randomized controlled trials in the highest impact factor journals: The SCOTI score. Ann Intensive Care. 2021; 11:165 34837580 10.1186/s13613-021-00954-x PMC8626742

[5] Moher D Liberati A Tetzlaff J; PRISMA Group: Preferred reporting items for systematic reviews and meta-analyses: The PRISMA statement. BMJ. 2009; 339:b2535 19622551 10.1136/bmj.b2535 PMC2714657

[6] ClinCalc.com: Fragility Index calculator. 2018. Available at: https://clincalc.com/Stats/FragilityIndex.aspx. Accessed December 13, 2021

[7] Kampman JM Turgman O Sperna Weiland NH: Statistical robustness of randomized controlled trials in high-impact journals has improved but was low across medical specialties. J Clin Epidemiol. 2022; 150:165–170 35820586 10.1016/j.jclinepi.2022.07.001

[8] Potter GE: Dismantling the Fragility Index: A demonstration of statistical reasoning. Stat Med. 2020; 39:3720–3731 32781488 10.1002/sim.8689

[9] Baer BR Gaudino M Fremes SE: Reassembling the Fragility Index: A demonstration of statistical reasoning. J Clin Epidemiol. 2022; 142:317–318 34710563 10.1016/j.jclinepi.2021.10.010

[10] Abrams D Montesi SB Moore SKL: Powering bias and clinically important treatment effects in randomized trials of critical illness. Crit Care Med. 2020; 48:1710–1719 33031148 10.1097/CCM.0000000000004568 PMC7708428

[11] Aberegg SK Richards DR O’Brien JM: Delta inflation: A bias in the design of randomized controlled trials in critical care medicine. Crit Care. 2010; 14:R77 20429873 10.1186/cc8990 PMC2887200

