# Supplementary material for: Randomized Controlled Trials in ICU in the Four Highest-Impact General Medicine Journals
Source: Crit Care Med. 2023 May 18;51(9):e179–83. doi: 10.1097/CCM.0000000000005937 (PMC10426774; doi:10.1097/CCM.0000000000005937)
Supplement: Supplementary file 1 [file ccm-51-e179-s001.docx]

**Supplementary Content**

*Title:* Randomized controlled trials in intensive care medicine in ‘the big four’ general medicine journals

*Authors:* Jasper M. Kampman, Niek H. Sperna Weiland, Jeroen Hermanides, Markus W. Hollmann, Sjoerd Repping, Janneke Horn

**Table of contents**

**Appendix 1: Search strategy page 2**

**Appendix 2: Exclusion criteria page 3**

**Appendix 3: Collected characteristics page 4**

**Appendix 4: List of ICU RCTs included in the Fragility Index analysis page 5**

**Appendix 1: Search strategy**

**Top 4 Journals by impact factor**

Source: Web of Science, InCites Journal Citation Reports, Year 2020 sorted by Impact Factor.

Top 4 in General Medicine

1. New England Journal of Medicine 91.253
2. Lancet 79.323
3. JAMA 56.274
4. British Medical Journal 39.890

**PubMed search (14-10-2021, results 2770)**

(“The New England journal of medicine”[Journal] OR “Lancet (London, England)”[Journal] OR “JAMA”[Journal] OR “BMJ (Clinical research ed.)”[Journal])

AND

(“2014/01/01”[Date – Publication] : “2021/09/30”[Date – Publication])

AND

("Randomized Controlled Trial" [Publication Type] OR randomized controlled trial[tiab] OR randomised controlled trial[tiab])

**Appendix 2: Exclusion criteria**

**List of exclusion criteria**

- Follow-up of previously reported initial RCT
- Non-randomized trial
- Additional analysis of data from a primary RCT
- Interim analysis
- No patient care involved
- Correction notices
- Duplicates
- Retraction notices
- Dose-finding studies without a placebo group
- Protocol publication of an RCT
- Retracted articles

**Appendix 3: Collected characteristics**

**List of characteristics collected from the included RCTs**

- Journal of publication
- Year of publication
- Sample size (at randomization)
- Study outcome
  - Positive (i.e. reached statistical significance for the primary outcome)
  - Negative (i.e. did not reach statistical significance for the primary outcome)
- Study design
  - Superiority
  - Non-inferiority
- Type of funding
  - Commercial
  - Non-commercial
  - Both
  - Neither
- Type of intervention
  - Medicine, food, fluids or supplements
  - Device, material (e.g. using an extracorporeal membrane oxygenation device, or using an impregnated central venous catheter to prevent infections)
  - Surgery or procedure
  - Behavioral or educational intervention, or physical training
  - Organizational intervention (e.g. timing of renal replacement therapy, or the initiation of a systematic ICU triage for critically ill elderly patients)
  - Diagnostic intervention

**Appendix 4: List of ICU RCTs included in the Fragility Index analysis**

| Authors | Title | Year | Journal | Fragility Index | Sample size | Fragility Quotient | Outcome measure |
| --- | --- | --- | --- | --- | --- | --- | --- |
| J. J. Parienti, N. Mongardon, B. Megarbane, et al. | Intravascular Complications of Central Venous Catheterization by Insertion Site | 2015 | NEJM | 5 | 3471 | 0,000864 | Composite: bloodstream infection, deep venous thrombosis |
| T. Fivez, D. Kerklaan, D. Mesotten, et al. | Early versus Late Parenteral Nutrition in Critically Ill Children | 2016 | NEJM | 29 | 1440 | 0,020139 | Composite: infection during ICU stay, duration of ICU stay |
| R. E. Gilbert, Q. Mok, K. Dwan, et al. | Impregnated central venous catheters for prevention of bloodstream infection in children (the CATCH trial): a randomised controlled trial | 2016 | The Lancet | 1 | 1485 | 0,000673 | Bloodstream infections |
| M. Girardis, S. Busani, E. Damiani, et al. | Effect of Conservative vs Conventional Oxygen Therapy on Mortality Among Patients in an Intensive Care Unit: The Oxygen-ICU Randomized Clinical Trial | 2016 | JAMA | 3 | 434 | 0,006912 | ICU mortality |
| G. Hernandez, C. Vaquero, P. Gonzalez, et al. | Effect of Postextubation High-Flow Nasal Cannula vs Conventional Oxygen Therapy on Reintubation in Low-Risk Patients: A Randomized Clinical Trial | 2016 | JAMA | 6 | 527 | 0,011385 | Reintubation rate <72 hours |
| S. Jaber, T. Lescot, E. Futier, et al. | Effect of Noninvasive Ventilation on Tracheal Reintubation Among Patients With Hypoxemic Respiratory Failure Following Abdominal Surgery: A Randomized Clinical Trial | 2016 | JAMA | 2 | 293 | 0,006826 | Reintubation rate <7 days |
| B. K. Patel, K. S. Wolfe, A. S. Pohlman, et al. | Effect of Noninvasive Ventilation Delivered by Helmet vs Face Mask on the Rate of Endotracheal Intubation in Patients With Acute Respiratory Distress Syndrome: A Randomized Clinical Trial | 2016 | JAMA | 10 | 83 | 0,120482 | Intubation rate |
| X. Su, Z. T. Meng, X. H. Wu, et al. | Dexmedetomidine for prevention of delirium in elderly patients after non-cardiac surgery: a randomised, double-blind, placebo-controlled trial | 2016 | The Lancet | 26 | 700 | 0,037143 | Delirium incidence |
| A. B. Cavalcanti, E. A. Suzumura, L. N. Laranjeira, et al. | Effect of Lung Recruitment and Titrated Positive End-Expiratory Pressure (PEEP) vs Low PEEP on Mortality in Patients With Acute Respiratory Distress Syndrome: A Randomized Clinical Trial | 2017 | JAMA | 0 | 1010 | 0 | 28-day mortality |
| D. Annane, A. Renault, C. Brun-Buisson, et al. | Hydrocortisone plus Fludrocortisone for Adults with Septic Shock | 2018 | NEJM | 3 | 1241 | 0,002417 | 90-day mortality |
| G. D. Perkins, C. Ji, C. D. Deakin, et al. | A Randomized Trial of Epinephrine in Out-of-Hospital Cardiac Arrest | 2018 | NEJM | 6 | 8014 | 0,000749 | 30-day mortality |
| M. W. Semler, W. H. Self, J. P. Wanderer, et al. | Balanced Crystalloids versus Saline in Critically Ill Adults | 2018 | NEJM | 0 | 15802 | 0 | Composite: death, renal-replacement therapy, renal dysfunction |
| Thille AW, Muller G, Gacouin A, et al. | Effect of Postextubation High-Flow Nasal Oxygen With Noninvasive Ventilation vs High-Flow Nasal Oxygen Alone on Reintubation Among Patients at High Risk of Extubation Failure: A Randomized Clinical Trial | 2019 | JAMA | 3 | 648 | 0,00463 | Reintubation rate day 7 |
| Subirà C, Hernández G, Vázquez A, et al. | Effect of Pressure Support vs T-Piece Ventilation Strategies During Spontaneous Breathing Trials on Successful Extubation Among Patients Receiving Mechanical Ventilation: A Randomized Clinical Trial | 2019 | JAMA | 21 | 1153 | 0,018213 | Reintubation rate |
| François B, Cariou A, Clere-Jehl R, et al. | Prevention of Early Ventilator-Associated Pneumonia after Cardiac Arrest | 2019 | NEJM | 2 | 198 | 0,010101 | VAP in first 7 ICU days |
| Lascarrou JB, Merdji H, Le Gouge A, et al. | Targeted Temperature Management for Cardiac Arrest with Nonshockable Rhythm | 2019 | NEJM | 1 | 584 | 0,001712 | Favorable neurologic outcome |
| Yannopoulos D, Bartos J, Raveendran G, et al. | Advanced reperfusion strategies for patients with out-of-hospital cardiac arrest and refractory ventricular fibrillation (ARREST): a phase 2, single centre, open-label, randomised controlled trial | 2020 | The Lancet | 1 | 30 | 0,033333 | Survival to hospital discharge |
| Milstone AM, Voskertchian A, Koontz DW, et al. | Effect of Treating Parents Colonized With Staphylococcus aureus on Transmission to Neonates in the Intensive Care Unit: A Randomized Clinical Trial | 2020 | JAMA | 2 | 236 | 0,008475 | Infection of neonate with S aureus strain of the parent by 90 days |
| Vourc'h M, Garret C, Gacouin A, et al. | Effect of High-Dose Baclofen on Agitation-Related Events Among Patients With Unhealthy Alcohol Use Receiving Mechanical Ventilation: A Randomized Clinical Trial | 2021 | JAMA | 1 | 314 | 0,003185 | Composite: 'agitation related events' |
